# Food safety practice and its associated factors among household food handlers in Patuakhali, Bangladesh: A cross-sectional study

**DOI:** 10.1371/journal.pone.0326595

**Published:** 2025-06-20

**Authors:** M. M. Mehedi Hasan, Nayeem Rahman, Md. Mofidul Islam, H. M. Sabbir Ahmed, Shuvajit Mondal, Farzana Afroz, Mohammad Abdulla Al Noman

**Affiliations:** 1 Department of Human Nutrition and Dietetics, Patuakhali Science and Technology University, Patuakhali, Bangladesh; 2 Department of Biochemistry and Food Analysis, Patuakhali Science and Technology University, Patuakhali, Bangladesh; 3 Faculty of Arts, Social Sciences and Humanities, University of Wollongong, Wollongong, Australia; 4 Department of Food Technology and Engineering, Patuakhali Science and Technology University, Patuakhali, Bangladesh; University of Health and Allied Sciences, GHANA

## Abstract

Food safety practices play a crucial role in the prevention of foodborne diseases, particularly in low- and middle-income countries like Bangladesh. This study assessed food safety practices among household food handlers in Patuakhali, Bangladesh, and identified associated factors influencing these practices. A cross-sectional study was conducted among 300 randomly selected households, using structured interviews and direct observations. The findings revealed that only 46% of participants demonstrated good food safety practices, with notable deficiencies in proper handwashing techniques (36.7%). Multiple logistic regression analysis identified that secondary education (AOR = 2.84; 95% CI: 1.44, 5.59), government employment (AOR = 5.74; 95% CI: 1.24, 26.53), monthly income between 15,000 and 30,000 BDT (AOR = 4.50; 95% CI: 2.17, 9.31), and participation in food safety training (AOR = 5.01; 95% CI: 1.95, 12.90) were significantly associated with good food safety practices. Conversely, living in rural areas (AOR = 0.30; 95% CI: 0.13–0.67) and, being aged 39–58 years (AOR = 0.36; 95% CI: 0.15–0.84) were associated with poor food safety practices. Addressing these factors, particularly socioeconomic disparities and offering targeted food safety education, could significantly improve public health outcomes and overall food safety practices.

## Introduction

Food safety refers to the procedures and measures taken to ensure that food is handled, prepared, stored, and consumed in ways that prevent foodborne diseases (FBDs) [1]. FBDs are caused by a wide range of pathogens, including bacteria, viruses, and parasites, which contaminate food through improper handling, inadequate cooking, unsafe food storage, and poor hygiene practices [2]. According to the World Health Organization (WHO), each year, eating contaminated food causes 600 million cases of FBDs, affecting nearly 1 in 10 people, resulting in approximately 420,000 deaths globally [3]. These figures underscore the severity of FBDs and highlight the critical need for effective food safety practices. Beyond direct health consequences, FBDs impose substantial economic burdens, including increased healthcare costs, household financial strain, and higher government expenditures [4]. Low and middle-income countries are disproportionately affected, incurring approximately $110 billion annually in healthcare expenses due to FBDs [3]. An estimated 30 million people in Bangladesh alone suffer from FBDs each year [5]. Unsafe food handling practices play a significant role in their spread, with approximately 10–20% of outbreaks linked to contamination by food handlers [6]. These risks arise from inadequate hand hygiene, cross-contamination, improper food storage, and insufficient cleaning and sanitization of surfaces and utensils [7,8]. While such issues are well-documented in commercial food settings, household food handlers frequently neglect these critical safety measures, further increasing the risk of contamination at the domestic level. Studies have demonstrated that improper food handling in the household can significantly increase the likelihood of FBDs [10]. Therefore, food safety practices within the home are essential in reducing the risk of contamination and illness [9].

Several studies have examined food handling across different settings, including restaurants, street food, retail markets and the food industry in Ethiopia [10,11], Ghana [12,13] and Bangladesh [14,15]. A study by Tamiru et al. [16] reported that 44.9% of food handlers demonstrated poor food safety practices. Among them 92.7% did not check food temperatures, 74% failed to sanitize after sneezing, and 32.2% did not use separate utensils for raw and cooked foods. Another study revealed that food safety practices among mothers in Debarq Town are suboptimal, with 49.6% of participants reporting good food safety practices, while 50.4% reported poor practices and key factors associated with good food safety practices are secondary educational status, food safety knowledge and attitude towards food safety [17]. Islam et al. [14] reported that only 17.6% of domestic food handlers in Bangladesh demonstrated adequate food safety knowledge, reflecting a significant knowledge gap. Furthermore, there was a lack of knowledge about food handling, food poisoning, and food storage. However, rather than analyzing food safety practices, the self-reported survey mainly assessed knowledge about food handling, food storage, and personal cleanliness. Another study conducted on meat handlers in Bangladesh showed that only 16.3% of meat handles showed good food safety practices, where food safety knowledge and working hours were significantly associated with food safety practices [15]. Naeem et al. [18] assessed the food safety knowledge, attitude, and practices of 1,000 household women in Lahore, revealing that 91.1% had inadequate knowledge, 85.0% exhibited a negative attitude, and 99.7% practiced unhygienic food handling, highlighting the need for targeted educational interventions.

Bangladesh, with its dense population and high risk of FBD outbreaks, has a majority of its population relying on homemade food [19]. However, research on food safety practices among household food handlers remains limited. Most existing studies focus on commercial food handling in restaurants, street vendors, and food industries. A population that contributes the most to food preparation remains largely overlooked, leaving a critical gap in understanding household food safety practices and their potential impact on public health. Therefore, this study aims to evaluate food safety practices and identify associated factors among food handlers in Bangladeshi households.

## Methodology

### Study design, period, and area

A cross-sectional study was carried out between March and June 2023 among 300 households in three upazilas of the Patuakhali District, located in the south-central region of Bangladesh, adjacent to the Bay of Bengal. The district spans 3,220.15 km² and comprises eight upazilas. Three upazilas – Patuakhali Sadar, Kalapara, and Bauphal – were randomly selected for the study.

### Sampling technique

**Fig 1:**
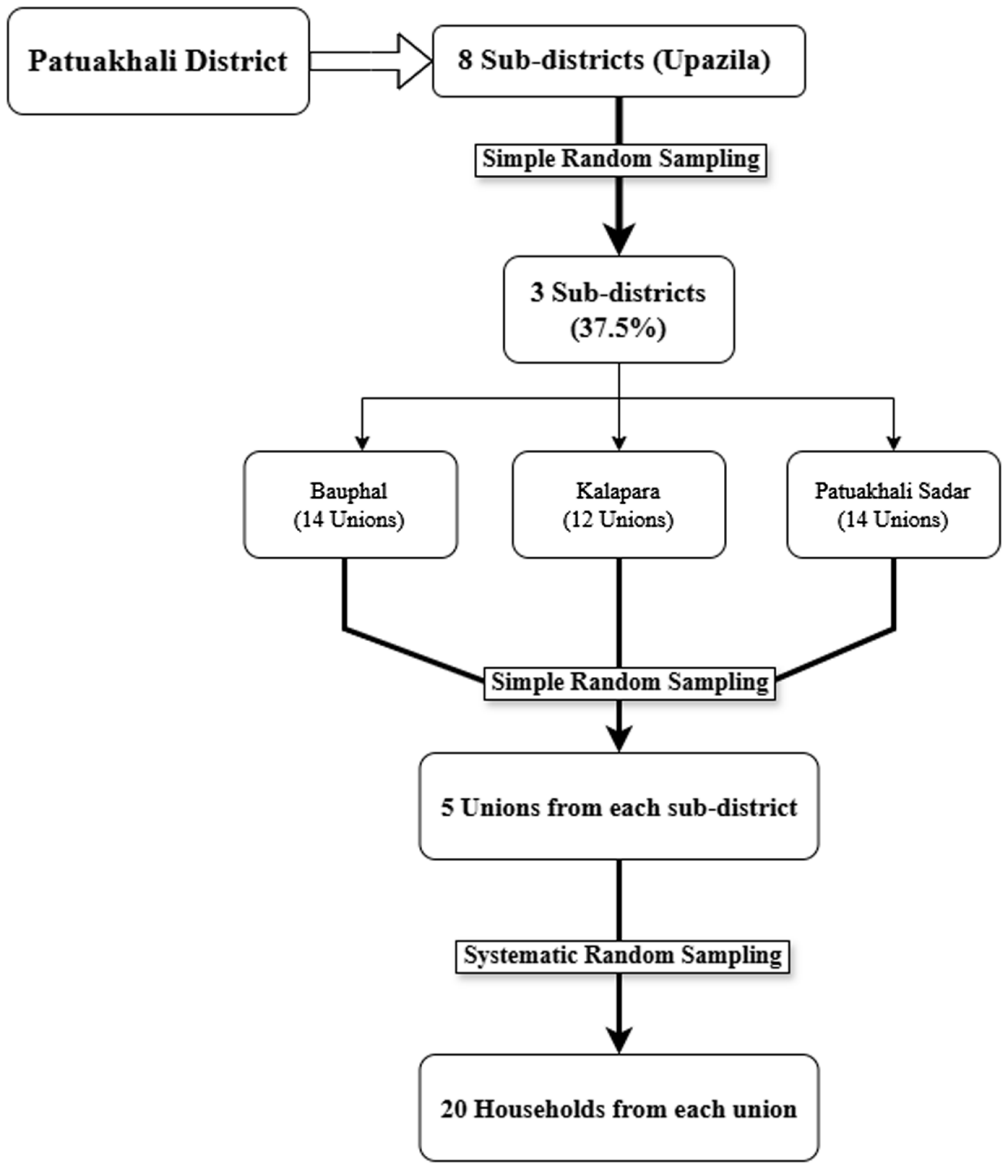
Multi-stage random sampling.

A multi-stage sampling approach was employed to ensure representative selection. First, three sub-districts were selected from the total eight sub-districts in the study area using simple random sampling. Each selected sub-district was further divided into unions, from which five unions were randomly selected using simple random sampling. Within each selected union, systematic sampling was applied to select 20 households. Enumerators selected a random starting point at a local market using a lottery method and followed a fixed interval walking pattern to systematically sample households. Since the total number of households was unknown, every 10^th^ household was selected as a sample household. If a selected household was unavailable or refused participation, the next nearest household was approached. This method enhances geographic coverage while minimizing selection bias, making it suitable for field-based surveys without a pre-existing household list (Fig 1).

### Sample size determination

The sample size for this study was initially calculated using Cochran’s formula, which is commonly employed for estimating proportions in large populations [20]. Based on a 95% confidence level, a 5% margin of error, and an assumed proportion of 50% (to maximize variability), the required sample size was determined to be 384 participants. However, due to recruitment challenges, 300 responses were obtained. While this reduction in sample size may slightly increase the margin of error and affect the representativeness of the sample, a post-hoc power analysis was conducted to assess the adequacy of the sample size. A post-hoc power analysis indicated a statistical power exceeding 98%, indicating that the sample size was sufficient to detect meaningful associations in food safety practices.

### Inclusion criteria and exclusion criteria

Female individuals who were primarily responsible for cooking in the household were considered as the sample for this study. In households where multiple women participated in food preparation, the primary respondent was identified as the individual with the most responsibility for food handling, as determined by self-reporting. We chose to focus on female individuals responsible cooking in the household as our sample population because they are the primary food handlers in Bangladeshi households. As the individuals most responsible for food preparation, storage, and overall kitchen management, they have the greatest influence on household food safety practices. Individuals who were seriously ill or who did not provide their consent were excluded from the study.

### Data collection tools and procedure

This study used a structured questionnaire adapted from previous study and modified to suit the study location and target participants [16,21,22]. The questionnaire was initially developed in English, translated into Bengali, and back-translated into English to ensure consistency. Data were gathered through face-to-face interviews and observation methods. Discrepancies were observed between participants’ self-reported practices and their actual behaviors [23], reinforcing the reliability of direct observation as a data collection method. To ensure consistency and accuracy in data collection, training was provided to all data enumerators before the fieldwork. The training sessions covered the standardized use of the observational checklist, proper identification of food safety practices, and strategies to minimize observer bias. Enumerators were instructed on maintaining objectivity and ensuring uniform assessment across different households.

The questionnaire was divided into 2 sections, consisting of 22 items. The first section focused on sociodemographic information and included 10 questions. The second section contained 12 questions designed to assess respondents’ food safety practices, 7 of which were based on observations.

Participants were asked to cooperate with the data enumerator by answering face-to-face questions and fulfilling the observational checklist. Participation was anonymous and voluntary. A pilot test involving 30 respondents was conducted to assess the questionnaire’s clarity and suitability.

### Operational definitions

#### Practice.

This section had 12 close-ended questions with two responses: “Yes” and “No.” Each correct practice reported scored 1 point. For evaluation, a score ≥ 70% by an individual respondent was considered as “good” food safety practice, whereas a score <70% was considered as “poor” food safety practice [11,12].

#### Right handwashing procedure.

Involves wetting hands with clean, running water and applying soap, followed by lathering thoroughly, ensuring coverage of the backs of the hands, between the fingers, and under the nails. Hands should be scrubbed for at least 20 seconds before being rinsed under clean, running water. Finally, they should be dried using a clean towel or by air drying [24].

### Data management and statistical analysis

Data were analyzed using Microsoft Excel and Statistical Package for the Social Science (SPSS) Software (version 27.0). Descriptive analysis was used to determine the percentage and number of participants’ distributions by socio-demographic characteristics. Bivariate analysis was conducted to explore the relationship between independent variables and food safety practices, subsequently multiple logistic regression analysis was performed to identify factors associated with food safety practices. Both univariate (unadjusted) and multivariable (adjusted) logistic regression models included all sociodemographic variables. All explanatory variables that were theoretically relevant or known from previous literature to influence food safety practices were considered for inclusion in the multivariable logistic regression model, regardless of their bivariate statistical significance. Bivariate significance is not a prerequisite for inclusion in multivariable analysis, as excluding important confounders may lead to biased estimates of association [25]. The Hosmer and Lemeshow test was used to check the model’s fitness, and the result (p = 0.343) shows that the model fits the data well. The independent variables were examined for multicollinearity by the Variance Inflation Factor (VIF). The highest VIF among all independent variables for the adjusted model was 1.564. Studies suggest that independent variables’ VIF of less than 10 is acceptable [26,27]. Adjusted odds ratios (AOR) with 95% confidence intervals (CI) and a p-value of <0.05 were used to identify the variables significantly associated with household food handlers’ food safety practices.

### Ethical approval

The study protocol was reviewed and approved by the Institutional Ethical Committee (IEC) of Patuakhali Science and Technology University, Bangladesh (ethical approval reference number: PSTU/IEC/2023/48(1)) before the data collection. All participants were informed of the study objectives and provided written informed consent for data usage and publication. Confidentiality and anonymity were strictly maintained.

## Results

### Sociodemographic characteristics of food handlers

The study participants’ ages ranged from 18 to 58 years, with the highest proportion (42.7%) in the 29–38 age group. The majority (88.3%) of the participants were Muslims. Of the participants, 45.3% of participants had secondary education. The majority (87.0%) of the respondents were housewives. More than half (53.3%) of the respondents earned 15,000–30,000 BDT monthly (approximately 125–250 USD). The majority (87.3%) of the respondents had a nuclear family, and most (81.7%) of them lived in the rural area. Only 13.3% of the study participants attended food safety training (**Table 1**).

**Table 1:** Socio-demographic characteristics of study participants (n = 300).

Characteristics	Frequency (N = 300)	Percentage (%)
**Gender**		
Female	300	100.0
**Age (in years)**		
18-28	59	19.7
29-38	128	42.7
39 - 58	113	37.7
**Religion**		
Islam	265	88.3
Hindu	35	11.7
**Education Qualification**		
Primary or No Education	102	34.0
Secondary Education	136	45.3
Higher Secondary or Above	62	20.7
**Respondents Occupation**		
Housewife	261	87.0
Govt. Job	22	7.3
Non-govt job	17	5.7
Monthly Income, BDT*		
<15000 BDT	118	39.3
15000-30000 BDT	160	53.3
>30000 BDT	22	7.3
**Type of the Family**		
Nuclear	262	87.3
Joint	38	12.7
**Living Area**		
Sub-urban	55	18.3
Rural	245	81.7
**Attended any food safety training**		
Yes	40	13.3
No	260	86.7

*15,000 Bangladeshi Taka (BDT) equals to 125 USD.

### Food safety practice of food handlers

Most participants cleaned kitchen dirt regularly (80.0%) and had proper food storage equipment (73.7%), but only 57.0% washed hands before touching cooked food, 67.0% used separate utensils for raw and cooked food, 52.3% cover their hair, and 45.0% did not trim their nails. Of the 300 participants, 279 (93.0%) wash their hands before preparing food and almost all wash raw food before use (98.7%). But only 110 (36.7%) of them practice right handwashing procedure (**Table 2**).

**Table 2:** Food safety practices while food preparation, cooking & storage among household food handlers (n = 300).

Safety Practices	Category	Frequency	Percentage
**1. Practice the right Handwashing procedure**			
	No	190	63.3
	Yes	110	36.7
**2. Shorten/trim fingernails**			
	No	135	45.0
	Yes	165	55.0
3. Wash hands before food preparation and cooking			
	No	21	7.0
	Yes	279	93.0
4. Wash raw food before use			
	No	4	1.3
	Yes	296	98.7
5. Wash hands before touching cooked food			
	No	129	43.0
	Yes	171	57.0
6. Cover hair when preparing food			
	No	157	47.7
	Yes	143	52.3
**7. Use separate utensils for raw and cooked food**			
	No	99	33.0
	Yes	201	67.0
8. Clean utensils before preparing another item in case of single use			
	No	54	18.0
	Yes	246	82.0
**9. Cover the cooked food while stored**			
	No	53	17.7
	Yes	247	82.3
**10. Have the proper storage equipment (e.g., refrigerator)**			
	No	79	26.3
	Yes	221	73.7
**11. Use the bin for waste management**			
	No	131	43.7
	Yes	169	56.3
**12. Clean the kitchen dirt/rubbish regularly**			
	No	60	20
	Yes	240	80

*Observational Questions are marked as bold.

### Level of food safety practice

**Fig 2:**
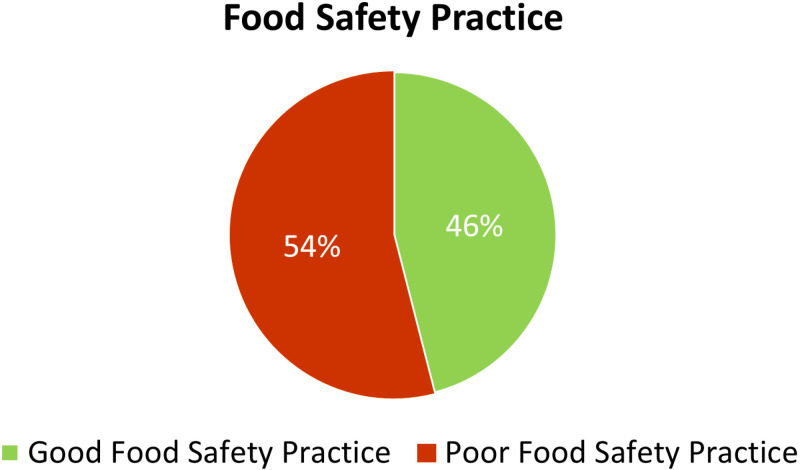
Overall food safety practices of household food handlers in Patuakhali, Bangladesh (n = 300).

Among all participants, 138 (46.0%) demonstrated good food safety practices, while 162 (54.0%) exhibited poor food safety practices (Fig 2).

### Factors associated with food safety practice

The association between sociodemographic characteristics and food safety practices is summarized in Table 3. The results of the chi-square tests revealed that food safety practices were significantly associated with age (p < 0.001), educational qualification (p < 0.001), occupation (p = 0.007), income level (p < 0.001), living area (p < 0.001), and food safety training (p < 0.001) (**Table 3**).

**Table 3:** Factors associated with food safety practice among household food handlers (n = 300).

Variable	Food Safety Practice		P – value^a^	Odds Ratio (95% CI)		VIF
	Poor	Good	Unadjusted	Adjusted^b^	
**Age (year)**						1.313
18–28	23	36	<0.001	Ref	Ref	
29–38	52	76		0.93 (0.49, 1.75)	1.42 (0.67, 3.03)	
39 - 58	87	26		0.19 (0.09, 0.37)***	0.36 (0.15, 0.84)*	
**Educational Qualification**						1.564
No Education or Primary	76	26	<0.001	Ref	Ref	
Secondary Education	61	75		3.59 (2.00, 6.28)*	2.84 (1.44, 5.59)**	
Higher Secondary or above	25	37		4.32 (2.20, 8.49)*	1.58 (0.62, 4.03)	
**Religion**						1.074
Islam	141	124	0.449	Ref	Ref	
Hindu	21	14		0.75 (0.37, 1.55)	0.37 (0.14, 0.94)*	
**Occupation**						1.406
Housewife	149	112	0.007	Ref	Ref	
Govt. Job	5	17		4.52 (1.62, 12.62)**	5.74 (1.24, 26.53)*	
Non-Govt. Job	8	9		1.49 (0.56, 4.00)	0.43 (0.12, 1.56)	
**Monthly Income**, BDT						1.300
Below 15000	56	17	<0.001	Ref	Ref	
15000–30000	95	110		3.81 (2.07, 7.00)***	4.50 (2.17, 9.31)***	
>30000	11	11		3.29 (1.21, 8.92)*	0.77 (0.16, 3.69)	
**Living Area**						1.146
Sub – Urban	16	39	<0.001	Ref	Ref	
Rural	146	99		0.27 (0.14, 0.52)***	0.30 (0.13, 0.67)**	
**Type of Family**						1.156
Nuclear	140	122	0.606	Ref	Ref	
Joint	22	16		0.83 (0.41, 1.66)	0.55 (0.21, 1.42)	
**Attend Any Food Safety Training**						1.181
No	152	108	<0.001	Ref	Ref	
Yes	10	30		4.22 (1.98, 9.00)***	5.01 (1.95, 12.90)***	

* Statistically significant at p < 0.05, ** Statistically significant at p < 0.01, *** Statistically significant at p < 0.001.

^a^ Calculated using the Chi-square analysis.

^b^ In this model, all the variables included in unadjusted model were adjusted.

The result of multiple logistic regression analysis is displayed in Table 3. Adjusted regression analysis showed that the odds of having good food safety practices are 2.84 times higher in participants with secondary education compared to those with primary or no education (AOR = 2.84; 95% CI: 1.44, 5.59). Individuals in government jobs have 5.74 times higher probability of good food safety practices compared to housewives (AOR = 5.74; 95% CI: 1.24, 26.53). The level of good food safety practice among participants who have a monthly income of 15000–30000 BDT (approximately 125–250 USD) is 4.5 times higher than that of those earning less than 15000 BDT (AOR = 4.50; 95% CI: 2.17, 9.31). Participants living in rural areas had significantly lower odds (by 70%) of practicing good food safety compared to those in suburban areas (AOR = 0.30; 95% CI: 0.13, 0.67). Those who attended food safety training were 5.01 times more likely to exhibit good food safety practices than those who did not (AOR = 5.01; 95% CI: 1.95, 12.90). Additionally, participants aged 39–58 years were 64% less likely to have good food safety practices compared to those aged 18–28 (AOR = 0.36; 95% CI: 0.15, 0.84).

## Discussion

This study identified key factors associated with food safety practices among household food handlers in Patuakhali, Bangladesh, using face-to-face interviews and direct observation. We found that 46.0% of participants demonstrated good safety practices. Similar levels were reported in studies from Ethiopia. Dagne et al. [17] reported a 49.6% prevalence of good food safety practices among mothers in Debarq Town, using a 12-item self-reported questionnaire, with scores above the mean indicating good practice. Keleb et al. [22] found that overall 44.7% household food handlers of Northeastern Ethiopia exhibited good food safety practices, assessed through a combination of interviews and observation. Similarly, Azanaw et al. [28] found a 49.0% prevalence among institutional food handlers in Gondar City, assessed through a 17-item questionnaire where scores above the mean denoted good food safety behavior. The observed similarities across these studies may be attributed to the shared socio-economic and cultural contexts typical of low-resource settings.

Although few studies in Bangladesh have directly assessed household food safety practices, a recent study by Islam et al. [14] reported that only 17.6% of household food handlers passed a food safety knowledge test. While their assessment focused solely on knowledge-based questions, not actual practices, their findings still highlight the consistently low level of food safety knowledge at the household level. Another study among meat handlers in Bangladesh found that only 16.3% demonstrated good food safety practices, with strong associations between education, training, and safe behavior – factors similarly identified in our study [15].

However, the proportion of participants with good food safety practices in our study (46.0%) was lower than reported in studies from Southwest Ethiopia (55.1%) [16], Ghana (62.9%) [13], Sri Lanka (59.5%) [29], Malaysia (77.7%) [30], Jordan (75.1%) [31], Nigeria (92.1%) [32]. The variation in results may be attributed to differences in the demographic characteristics of the study population or the methodological approach. Several of these studies involved institutional or occupational food handlers in urban settings [13,16,29,30], where individuals are more likely to have access to food safety training, infrastructure, and routine supervision. In contrast, our study focused on female household food handlers in a rural, disaster-prone area, where such support systems are often lacking.

Furthermore, many of the higher-scoring studies relied solely on self-reported data [31–33], which tend to overestimate adherence to safe practices. Our study, by comparison, utilized direct observational assessments for key behaviors, providing a more conservative but likely more accurate measure of actual food safety practices. This may partially explain the lower proportion observed in our findings.

The value of using observation over self-report is supported by prior research. For example, a study conducted in China among 900 rural consumers found that self-reported food safety behaviors were significantly better than observed behaviors, with males, elderly individuals, and highly educated consumers showing the largest discrepancies [23]. Similarly, a study conducted among Puerto Rican women preparing meals at home found significant discrepancies between self-reported and observed food safety behaviors. Socially desirable practices, such as handwashing and cutting board cleaning, were frequently over-reported, raising concerns about the reliability of self-reported data [34]. Another study conducted in rural Malawi similarly found that self-reported food hygiene practices were more frequently reported than observed [35]. These findings highlight the limitations of self-reported data and underscore the importance of observational methods in evaluating food handling practices.

Our study found that most of the participants wash hands before food preparation (93%), but when we asked them to wash their hands, we found that only 37% of participants followed the proper handwashing procedure recommended by the Centers for Disease Control and Prevention (CDC) [24]. This is a major concern, as pathogens can still spread if proper handwashing procedures are not followed.

Results from multiple logistic regression analysis revealed that education, occupation, monthly income, and food safety training were significantly associated with good food safety practices. The likelihood of having better food safety practices was higher among those who attended food safety training than those who did not. A similar result was found in previous studies conducted in Bangladesh [15], Saudi Arabia [36], Northeastern Ethiopia [22], Debarq town [17], Gondar City [28], Abobo District [37] and Ghana [13]. Improved food safety practices and food safety training have been consistently found to be strongly correlated in numerous studies. Training empowers people to understand potential risks and take precautions, including hygiene maintenance, safe food handling, and proper storage. Investing in food safety training programs can significantly enhance household-level food handling practices and reduce foodborne diseases risks.

Food safety practices were also significantly influenced by education. Participants with more educational qualifications, especially those with a secondary education, showed significantly better food safety practices than those with only primary education or no formal education at all. This finding is consistent with earlier research that emphasize the importance of education in food safety practices [17,38–40]. However, it is interesting to note that although secondary education showed significantly better practices, the effect was not statistically significant with higher secondary education or above. This finding suggests that a certain level of basic education – particularly up to the secondary level – may be sufficient to instill essential knowledge and awareness regarding food safety practices. Secondary education often includes fundamental lessons on hygiene, health, and environmental science, which likely contribute to better understanding and adherence to food safety principles. In contrast, higher education beyond the secondary level may not necessarily reinforce these practices further, as food safety knowledge may not be a primary focus in advanced education curricula. Additionally, individuals with higher education may be engaged in occupations that reduce their direct involvement in household food handling, leading to less impact on their personal food safety practices. This trend has been observed in other studies, where basic education levels were found to be crucial for promoting hygiene and health-related behaviors, while higher education did not necessarily lead to additional improvements [17]. These findings highlight the importance of integrating food safety education into school curricula at the fundamental levels to ensure broader public health benefits.

In addition to education, occupation also played a significant role in food safety practices, with individuals employed in government jobs demonstrating significantly better practices compared to housewives. This might be because government jobs often provide training and emphasize hygiene at work, which helps build good habits. On the other hand, housewives, despite being the primary food handlers in households, often rely on traditional knowledge or informal learning, which may not always concur with scientifically recommended food safety practices. To address this gap, targeted interventions such as community-based workshops and home-based training programs should be prioritized for housewives and individuals in informal occupations.

Our findings indicate that household food handlers from middle-income households (BDT 15,000–30,000/month) were significantly more likely to practice good food safety measures compared to those from low-income households (below BDT 15,000/month). Financial stability enables families to access clean water, appropriate food storage containers, and higher-quality food, all of which contribute to improved food safety practices. Conversely, financial constraints make it more challenging to prioritize food safety. Providing essential resources such as safe storage containers and hygiene kits – including liquid hand soap, hand sanitizer, and dishwashing liquid – to low-income families can significantly enhance food safety practices. Additionally, microfinance or government assistance programs could support low-income families in acquiring essential kitchen infrastructure, such as kitchen shelves and refrigerators to improve food safety. However, this finding is inconsistent with previous studies conducted on mothers in Northwest Ethiopia [17,38]. This could be because the study might refer to the mother’s income, while our study considers household income.

The study’s findings on the factors linked to poor safety practices show that participants aged 39–58 years exhibit significantly poor safety practices than those in other age groups. This could be due to the lack of targeted educational intervention addressing the specific needs of older populations. Implementing proper educational programs tailored to older people should be introduced, focusing on easy-to-understand visual and practical demonstrations of safe food handling. Other studies suggested similar interventions for older people [41,42]. Additionally, establishing peer-led initiatives where trained community health workers or younger family members can educate elderly individuals on updated food safety guidelines could be an effective approach.

Furthermore, participants in suburban areas demonstrated significantly better food safety practices compared to those in rural areas. This difference might be attributed to better infrastructure, greater exposure to health campaigns, and access to information in sub-urban settings [43,44]. Suburban areas generally have better access to clean water, electricity, proper food storage facilities (such as refrigerators), and sanitation services, which facilitate improved food safety practices. Additionally, residents in suburban settings are more likely to be exposed to public health campaigns and educational programs through local healthcare centers, schools, and media outlets. Government and non-governmental organizations often implement food safety and hygiene awareness programs in semi-urban areas where access to organized training sessions is more feasible. In contrast, rural areas may face limitations in these aspects. Households often rely on traditional food handling practices, which may not always align with scientifically recommended safety measures. Limited access to structured food safety training and fewer health outreach programs in rural communities contribute to the persistence of unsafe food handling behaviors.

To improve food safety practices in rural areas, targeted interventions should focus on expanding access to food safety education through community health workers, mobile health units, and local agricultural extension programs. Leveraging widely used communication channels such as radio, social media, and community-based workshops can help bridge the knowledge gap. Additionally, policies aimed at improving rural infrastructure, such as access to clean water and electricity for food preservation, could further support better food safety practices. Strengthening these factors could significantly contribute to reducing disparities in food safety practices between suburban and rural populations.

### Strengths and limitations

A major strength of this study is the use of both direct observations and interviews, reducing reliance on self-reported data and enhancing reliability. Another strength is the rigorous analysis, which enhances the accuracy and reliability of the findings. Our findings may be crucial for developing effective interventions and policies aimed at improving food safety practices. However, certain limitations must be acknowledged. One limitation of this study was the sample size. Although 384 was the calculated sample size, only 300 responses were obtained. This shortfall may have slightly increased the margin of error and affected the generalizability of the findings. Future research could aim to recruit larger sample sizes to strengthen the robustness of findings. Additionally, the study was conducted in a specific region (Patuakhali, Bangladesh) and focused exclusively on female household food handlers, as they represent the primary individuals responsible for food preparation in rural and semi-urban households in Bangladesh, which may limit the generalizability of the results to other settings.

## Conclusions

This study demonstrates that household food safety practices are significantly influenced by education, occupation, income, and training. Individuals with higher education, stable employment (particularly in government roles), and middle-income levels were more likely to follow proper food safety practices, likely because they have better access to resources and information. Conversely, individuals with lower income or education faced greater barriers, such as limited access to food safety knowledge and resources, making it harder to maintain safe practices.

While education, income, and training have been previously associated with better food safety practices, this study adds new evidence by focusing specifically on female household food handlers in rural coastal Bangladesh, a group that is underrepresented in the literature. Moreover, by combining self-reported responses with direct observation, this study provides a more accurate picture of actual food handling behaviors. These findings highlight the urgent need for context-specific, community-based interventions that consider local socioeconomic realities and cultural norms. Expanding food safety training tailored to rural women who are the primary food handlers, and improving access to hygiene infrastructure, could lead to meaningful reductions in foodborne illnesses at the household level.

## Supporting information

S1 File Dataset. (CSV)

## References

[1] What Is Food Safety? A Complete Guide for Food Businesses. [cited 21 Dec 2024]. https://blog.foodsafety.com.au/what-is-food-safety

[2] Augustin J-C, Kooh P, Bayeux T, Guillier L, Meyer T, Jourdan-Da Silva N, et al. Contribution of Foods and Poor Food-Handling Practices to the Burden of Foodborne Infectious Diseases in France. Foods. 2020;9(11):1644. doi: 10.3390/foods9111644 33187291 PMC7697675

[3] Food safety. [cited 17 Mar 2025]. https://www.who.int/news-room/fact-sheets/detail/food-safety

[4] Jaffee S, Henson S, Unnevehr L, Grace D, Cassou E. The Safe Food Imperative: Accelerating Progress in Low- and Middle-Income Countries. Washington, DC: World Bank. 2018. doi: 10.1596/978-1-4648-1345-0

[5] Khairuzzaman M, Chowdhury FM, Zaman S, Al Mamun A, Bari ML. Food Safety Challenges towards Safe, Healthy, and Nutritious Street Foods in Bangladesh. Int J Food Sci. 2014;2014:483519. doi: 10.1155/2014/483519 26904635 PMC4745531

[6] Mudey AB, Kesharwani N, Mudey GA, Goyal RC, Dawale AK, Wagh VV. Health Status and Personal Hygiene among Food Handlers Working at Food Establishment around a Rural Teaching Hospital in Wardha District of Maharashtra, India. GJHS. 2010;2(2). doi: 10.5539/gjhs.v2n2p198

[7] Chen Y, Wan G, Song J, Dai J, Shi W, Wang L. Food Safety Practices of Food Handlers in China and their Correlation with Self-reported Foodborne Illness. J Food Prot. 2024;87(1):100202. doi: 10.1016/j.jfp.2023.100202 38052368

[8] Tappes SP, Chaves Folly DC, da Silva Santos G, de Aquino Feijó C, Pustiglione M. Food handlers and foodborne diseases: grounds for safety and public and occupational health actions. Rev Bras Med Trab. 2020;17(3):431–40. doi: 10.5327/Z1679443520190316 32368677 PMC7195885

[9] Langiano E, Ferrara M, Lanni L, Viscardi V, Abbatecola AM, De Vito E. Food safety at home: knowledge and practices of consumers. Z Gesundh Wiss. 2012;20(1):47–57. doi: 10.1007/s10389-011-0437-z 22347771 PMC3268974

[10] Abdi AM, Amano A, Abrahim A, Getahun M, Ababor S, Kumie A. Food Hygiene Practices and Associated Factors Among Food Handlers Working in Food Establishments in the Bole Sub City, Addis Ababa, Ethiopia. Risk Manag Healthc Policy. 2020;13:1861–8. doi: 10.2147/RMHP.S266342 33061719 PMC7535140

[11] Gebru SB, Hailu TS, Taffere GR. Food Safety Knowledge, Attitude, and Practice of Food Handlers at Food Service Establishments in Southern Tigray, Ethiopia. Glob Soc Welf. 2023;10(3):249–62. doi: 10.1007/s40609-023-00284-9

[12] Akabanda F, Hlortsi EH, Owusu-Kwarteng J. Food safety knowledge, attitudes and practices of institutional food-handlers in Ghana. BMC Public Health. 2017;17(1):40. doi: 10.1186/s12889-016-3986-9 28061850 PMC5219779

[13] Tuglo LS, Agordoh PD, Tekpor D, Pan Z, Agbanyo G, Chu M. Food safety knowledge, attitude, and hygiene practices of street-cooked food handlers in North Dayi District, Ghana. Environ Health Prev Med. 2021;26(1):54. doi: 10.1186/s12199-021-00975-9 33941082 PMC8091506

[14] Islam MdN, Roy N, Amin MdB, Madilo FK, Karmakar K, Hossain E, et al. Food safety knowledge and handling practices among household food handlers in Bangladesh: A cross-sectional study. Food Control. 2023;147:109578. doi: 10.1016/j.foodcont.2022.109578

[15] Al Banna MH, Disu TR, Kundu S, Ahinkorah BO, Brazendale K, Seidu A-A, et al. Factors associated with food safety knowledge and practices among meat handlers in Bangladesh: a cross-sectional study. Environ Health Prev Med. 2021;26(1):84. doi: 10.1186/s12199-021-01004-5 34454422 PMC8403395

[16] Tamiru S, Bidira K, Moges T, Dugasa M, Amsalu B, Gezimu W. Food safety practice and its associated factors among food handlers in food establishments of Mettu and Bedelle towns, Southwest Ethiopia, 2022. BMC Nutr. 2022;8(1):151. doi: 10.1186/s40795-022-00651-3 36550561 PMC9773440

[17] Dagne H, Raju RP, Andualem Z, Hagos T, Addis K. Food Safety Practice and Its Associated Factors among Mothers in Debarq Town, Northwest Ethiopia: Community-Based Cross-Sectional Study. Biomed Res Int. 2019;2019:1549131. doi: 10.1155/2019/1549131 31275961 PMC6582849

[18] Naeem N, Raza S, Mubeen H, Siddiqui SA, Khokhar R. Food safety knowledge, attitude, and food handling practices of household women in Lahore. Journal of Food Safety. 2018;38(5). doi: 10.1111/jfs.12513

[19] Mottaleb KA, Rahut DB, Mishra AK. Consumption of food away from home in Bangladesh: Do rich households spend more?. Appetite. 2017;119:54–63. doi: 10.1016/j.appet.2017.03.030 28347779

[20] Cochran WG. Sampling techniques. 3 ed. New York: J. Wiley & sons. 1977.

[21] Negassa B, Anbese AT, Worku G, Areba AS, Seboka BT, Debela BG, et al. Food Hygiene Practices and Associated Factors Among Street Food Vendors in Urban Areas of Gedeo Zone, Southern Ethiopia. Environ Health Insights. 2023;17:11786302231168531. doi: 10.1177/11786302231168531 37122687 PMC10134189

[22] Keleb A, Ademas A, Sisay T, Adane M. Self-Reported Food Safety Practices and Associated Factors Among Health Extension Model and Non-Model Households in Northeastern Ethiopia: A Comparative Cross-Sectional Study. Risk Manag Healthc Policy. 2022;15:375–88. doi: 10.2147/RMHP.S353181 35283652 PMC8904758

[23] Zhang M, Zhu Q, Bai J. The disparity between self-reported and observed food safety behavior: A case involving consumers from rural China. Food Control. 2022;138:108981. doi: 10.1016/j.foodcont.2022.108981

[24] CDC. About Handwashing. In: Clean Hands [Internet]. 30 Apr 2024 [cited 15 Mar 2025]. https://www.cdc.gov/clean-hands/about/index.html

[25] Sun GW, Shook TL, Kay GL. Inappropriate use of bivariable analysis to screen risk factors for use in multivariable analysis. J Clin Epidemiol. 1996;49(8):907–16. doi: 10.1016/0895-4356(96)00025-x 8699212

[26] O’brien RM. A Caution Regarding Rules of Thumb for Variance Inflation Factors. Qual Quant. 2007;41(5):673–90. doi: 10.1007/s11135-006-9018-6

[27] Kim JH. Multicollinearity and misleading statistical results. Korean J Anesthesiol. 2019;72(6):558–69. doi: 10.4097/kja.19087 31304696 PMC6900425

[28] Azanaw J, Gebrehiwot M, Dagne H. Factors associated with food safety practices among food handlers: facility-based cross-sectional study. BMC Res Notes. 2019;12(1):683. doi: 10.1186/s13104-019-4702-5 31640793 PMC6805513

[29] Galgamuwa LS, Iddawela D, Dharmaratne SD. Knowledge and practices of food hygiene among food handlers in plantation sector, Sri Lanka. Int J Sci Rep. 2016;2(12):304. doi: 10.18203/issn.2454-2156.intjscirep20164307

[30] Abdullahi A, Hassan A, Kadarman N, Saleh A, Baraya YS, Lua PL. Food safety knowledge, attitude, and practice toward compliance with abattoir laws among the abattoir workers in Malaysia. Int J Gen Med. 2016;9:79–87. doi: 10.2147/IJGM.S98436 27110137 PMC4835135

[31] Alraei WY, Aljaraedah TY, El-Qudah JMF, Aljaraedah TY, Abu-Harirah HA, Rahahleh RJ, et al. Assessment of Food Safety, Knowledge and Practices among Students and Staff at Zarqa University, Jordan. TJNPR. 2023;7(5):2878–83. doi: 10.26538/tjnpr/v7i5.6

[32] Uzoama JO, Kimm H, Konlan KD. Factors associated with food safety practices among food handlers in Abuja municipal area council, Nigeria. J Glob Health Sci. 2023;5(1). doi: 10.35500/jghs.2023.5.e9

[33] Lee H, Abdul Halim H, Thong K, Chai L. Assessment of Food Safety Knowledge, Attitude, Self-Reported Practices, and Microbiological Hand Hygiene of Food Handlers. IJERPH. 2017;14(1):55. doi: 10.3390/ijerph14010055

[34] Dharod JM, Pérez-Escamilla R, Paciello S, Bermúdez-Millán A, Venkitanarayanan K, Damio G. Comparison between self-reported and observed food handling behaviors among Latinas. J Food Prot. 2007;70(8):1927–32. doi: 10.4315/0362-028x-70.8.1927 17803151

[35] Chidziwisano K, Tilley E, Morse T. Self-Reported Versus Observed Measures: Validation of Child Caregiver Food Hygiene Practices in Rural Malawi. Int J Environ Res Public Health. 2020;17(12):4498. doi: 10.3390/ijerph17124498 32585833 PMC7344643

[36] Alqurashi NA, Priyadarshini A, Jaiswal AK. Evaluating Food Safety Knowledge and Practices among Foodservice Staff in Al Madinah Hospitals, Saudi Arabia. Safety. 2019;5(1):9. doi: 10.3390/safety5010009

[37] Okugn A, Woldeyohannes D. Food hygiene practices and its associated factors among model and non model households in Abobo district, southwestern Ethiopia: Comparative cross-sectional study. PLoS One. 2018;13(4):e0194391. doi: 10.1371/journal.pone.0194391 29621267 PMC5886398

[38] Ayaz WO, Priyadarshini A, Jaiswal AK. Food Safety Knowledge and Practices among Saudi Mothers. Foods. 2018;7(12):193. doi: 10.3390/foods7120193 30477256 PMC6306806

[39] Iwu AC, Uwakwe KA, Duru CB, Diwe KC, Chineke HN, Merenu IA, et al. Knowledge, Attitude and Practices of Food Hygiene among Food Vendors in Owerri, Imo State, Nigeria. ODEM. 2017;05(01):11–25. doi: 10.4236/odem.2017.51002

[40] Meysenburg R, Albrecht JA, Litchfield R, Ritter-Gooder PK. Food safety knowledge, practices and beliefs of primary food preparers in families with young children. A mixed methods study. Appetite. 2014;73:121–31. doi: 10.1016/j.appet.2013.10.015 24211815

[41] Cates SC, Kosa KM, Karns S, Godwin SL, Speller-Henderson L, Harrison R, et al. Food Safety Knowledge and Practices among Older Adults: Identifying Causes and Solutions for Risky Behaviors. J Nutr Elder. 2009;28(2):112–26. doi: 10.1080/01639360902949986 21184361

[42] Berger N, Koch S, Jungnickel K, Böl G-F. Food safety in the aging population: Qualitative findings on what to communicate and how. Risk Anal. 2023;43(9):1843–54. doi: 10.1111/risa.14069 36368675

[43] Ishra R, Khanam R, Soar J. Influence of food safety concerns on safe food purchasing at rural and urban consumers in Bangladesh. Appetite. 2022;179:106306. doi: 10.1016/j.appet.2022.106306 36087826

[44] Adebowale O, Kassim IO. Food safety and health: a survey of rural and urban household consumer practices, knowledge to food safety and food related illnesses in Ogun State. ebph. 2022;14(3). doi: 10.2427/12568

